# The relationship of serum amylase levels in acute organophosphorus poisoning with its clinical severity and outcome: a cross-sectional study

**DOI:** 10.1097/MS9.0000000000000433

**Published:** 2023-04-06

**Authors:** Basudev Subedi, Gopal Kumar Yadav, Amar Raut, Nisha Joshi, Bal Krishna Subedi, Nimesh Joshi, Ram Prasad Neupane, Ved Bhandari, Ramesh Kumar Maharjan, Ramesh Prasad Acharya

**Affiliations:** Departments of a General Practice and Emergency Medicine; b Orthopedics; c Maharajgunj Medical Campus, Tribhuwan University Teaching Hospital, Kathmandu; d Department of Internal Medicine, Kalaiya Hospital, Bara; e Department of Emergency Medicine, Kakani Primary Health Care Center, Nuwakot, Nepal

**Keywords:** amylase, organophosphate, poisoning, prognostic marker

## Abstract

There are limited literatures studying the pancreatic involvement in organophosphate (OP) poisoning using biochemical means. This study focused on assessing the type of OP poisoning and determining the association of serum amylase levels with the patient’s presentation and outcome.

**Materials and methods:**

A cross-sectional study was carried out in the Maharajgunj Medical Campus, Tribhuwan University Teaching Hospital, Kathmandu, Nepal, after ethical approval [Ref: IRB/308 (6-11-E)]. We collected data from 172 participants with OP poisoning over the period of 2 years using nonprobability purposive sampling method. All patients with age group 16–75 years having a history of OP poisoning within the previous 24 h with clinical features and physical evidence of poisoning were included in the study. Those participants with indications of exposure to an entirely different poisons, poisoning with multiple poisons, OP poisoning along with alcohol, chronic alcoholics, comorbid conditions, taking drugs that could affect serum amylase levels (azathioprine, thiazides, furosemide, etc.), and/or treated in other hospitals after poisoning were excluded from the study. Appropriate statistical calculations were made using the statistical package for social sciences (SPSS), version 21. The *P*-value of less than 0.05 was considered statistically significant.

**Results:**

Metacid (53.5%, 92) was the most common OP poison. There were significantly higher mean values of serum amylase levels either within 12 h of exposure (468.60 vs. 135.4 IU/ml, *P*<0.001) or after 12 h of exposure (152.0 vs. 58.9 IU/ml, *P*<0.001) in dead participants than alive ones. The participants with initial and after 12 h of exposure-serum amylase level 100 or more IU/ml had more than two-fold and 18-fold higher odds of severe/life-threatening severity (odds ratio=2.40, 95% CI: 1.28–4.52, *P*=0.007 and odds ratio=18.67, 95% CI: 8.02–43.47, *P*<0.001) respectively than those with less than 100 IU/ml.

**Conclusions:**

The clinical severity of OP poisoning is directly related to serum amylase levels. Importantly, higher mean values of serum amylase levels were depicted in those participants with OP poisoning culminating to death. Thus, serum amylase level could be one of the easy measurable prognostic marker of OP poisonings.

## Introduction

Highlights

- The most common organophosphate (OP) compound used for poisoning is *Metacid.*
- Serum amylase could be an important prognostic marker of OP poisoning.
- This study demonstrated higher mean values of serum amylase levels among severe/life-threatening participants with OP poisoning.
- Higher mean values of serum amylase levels in participants with OP poisoning who became dead in the course of treatment suggests the outcome of participants could be gauzed based on serum amylase levels.

One of the commonest poisonings, that is, acute OP poisoning has attained epidemic proportion in most parts of the world. The toxicity of OP poison and lack of medical management especially in developing countries has led to its high fatality rate^1^,^2^. The incidence of OP as a self-poison is higher in younger active groups owing to their ease of access and sociocultural factors^3^.

Acute pesticide poisoning is a universal public health problem and accounts more than 0.3 million deaths^4^. The OP compounds hold a major proportions of inpatient deaths among poisoning cases in Nepal^5^,^6^. Thus, early recognition and timely intervention of toxicity from these compounds are of paramount importance. Case reports on acute pancreatitis following acute OP ingestion has been reported now and then, but appropriate literature with reference to the pancreatitis is not available. Hence, we attempted to study pancreatic involvement in OP poisoning biochemically.

Although we came across a large number of patients with this poisoning now in our clinical practice, there was no local literature available regarding the frequency of high amylase levels. In the study from India by Patil *et al*.^7^, hyperamylasemia was seen in all the cases with OP poisoning. This study was therefore designed to determine the type of OP used for poisoning and to assess the relationship of serum amylase levels with a patient’s presentation and outcome.

## Materials and methods

### Study design, sample size, and sampling technique

We carried out a cross-sectional study at the Maharajgunj Medical Campus, Tribhuwan University Teaching Hospital, Kathmandu, Nepal from 20 March 2017 to 19 March 2019. Since the national prevalence of OP poisoning was not available, 1-month emergency medical record was reviewed and the prevalence of OP poisoning was found to be 0.5% (17 August–16 September 2016). Assuming the prevalence of OP poisoning to be 0.5% with a significance level set at 5%, absolute precision of 1.05%, and a finite population correction, the sample size was computed to be 172. The nonprobability purposive sampling method was adopted for data collection. All patients with the age group 16–75 years having a history of OP poisoning within the previous 24 h with clinical features and physical evidence of poisoning were included in the study. Those participants with indications of exposure to an entirely different poison other than OP poison, poisoning with multiple poisons, OP poisoning along with alcohol, chronic alcoholics, comorbid conditions, taking drugs that could affect serum amylase levels (azathioprine, thiazides, furosemide, etc.), and/or treated in other hospitals after poisoning were excluded from the study. Bardin’s classification was adopted for gauzing the severity of the patients with OP poisoning^8^,^9^. Based on this, we categorized OP poisoning patients into either mild or severe/life-threatening groups. Mild OP poisoning groups included those with a history of exposure/intake with mild signs like normal consciousness, mild secretions, and little fasciculations. Similarly, severe/life-threatening OP poisoning patients included those with a history of intake/exposure with severe signs like altered consciousness, copious secretions, generalized fasciculations, suicide efforts, partial pressure of oxygen less than 75 mmHg, and abnormal roentgenogram. At least two criteria were required to grade. If fewer criteria are present, then the previous grade was used. This work has been registered with UIN researchregistry8675 and carried out in accordance with the STROCSS criteria^10^. The ethical clearance was obtained from the Institutional Review Committee of Maharajgunj Medical Campus, Tribhuwan University Teaching Hospital, Kathmandu, Nepal.

### Data entry and analysis

The data gathered were tabulated in Microsoft excel 2019, v16.0 (Microsoft), and analysis was carried out using the statistical package for social sciences (SPSS), version 11.5 (IBM SPSS, v22; IBM Corp.). Descriptive statistics like mean, SD, range, and/or percentages were used to present data like background characteristics and characteristics of poisoning products. The chi-square test was used to check the association between categories of initial and 12 h postexposure amylase levels with the patient’s severity. For binary logistic regression, odds ratio (OR) and 95% CI was calculated. For comparing mean serum amylase level between alive and dead patients within and after 12 h of exposure, an independent sample *t*-test was used. The *P*-value of less than 0.05 was regarded as statistically significant.

## Results

Of the 172 respondents, the mean age of participants was 34.6±14 years. The majority of the participants belonged to the age group 15–24 years (29.1%, 50) followed by 25–34 years (26.7%, 46). Similarly, the majority of the participants were female (74.4%, 128), and takes 30 min or more to reach the nearby hospital (74.4%, 129). About one-third of the participants (34.9%, 61) sought for help. The most common reason for poisoning was suicidal (97.7%, 168) (Table 1).

**Table 1:** Basic demographic characteristics of participants with organophosphate poisoning and their behavior

Characteristics	*N*=172 [*n* (%)]
Age (years)	
Mean±SD	34.6±14
Range	17–63
15–24	50 (29.1)
25–34	46 (26.7)
35–44	29 (16.9)
45–54	27 (15.7)
≥55	20 (11.6)
Sex	
Female	128 (74.4)
Male	44 (25.6)
Time to reach hospital (min)	
<30	43 (25.0)
≥30	129 (75.0)
Call for help	
Yes	61 (35.5)
No	111 (64.5)
Reason for poisoning	
Accidental	4 (2.3)
Suicidal	168 (97.7)

Among the different products available in the market, the common products were Metacid (53.5%, 92), Dichlorphos (14.0%, 24), and Defox (11.6%, 20). The common route of organophosphorus poisoning was ingestion (97.7%, 168). More than half of the participants (55.8%, 96) had taken less than 1 teaspoon full of poison (Table 2).

**Table 2:** Characteristics of poisoning products consumed by participants with organophosphate poisoning

Characteristics	*N*=172 [*n* (%)]
Product name	
Allethrin	4 (2.3)
Allout	4 (2.3)
Chlorpyriphos	8 (4.7)
Cypermethrin	8 (4.7)
Defox	20 (11.6)
Dichlorphos	24 (14.0)
Malathion	4 (2.3)
Metacid	92 (53.5)
Naryaw	4 (2.3)
Petes	4 (2.3)
Route	
Ingestion	168 (97.7)
Inhalation	4 (2.3)
Amount of poison	
<1 TSF	96 (55.8)
1 or >1 TSF	76 (44.2)

TSF, teaspoon full.

The participants with initial serum amylase (within 12 h of exposure) level 100 IU/ml or more had more than two-fold higher odds of severe/life-threatening severity (OR=2.40, 95% CI: 1.28–4.52, *P*=0.007) than those with less than 100 IU/ml. Similarly, the participants with serum amylase 100 IU/ml or more after 12 h of exposure had more than 18-fold higher odds of severe/life-threatening severity (OR=18.67, 95% CI: 8.02–43.47, *P*<0.001) than those with less than 100 IU/ml (Table 3).

**Table 3:** Relationship of serum amylase levels with clinical severity of participants with organophosphate poisoning (*N*=172)

	Patient’s severity [*n* (%)]			
Characteristics	Mild	Severe/life-threatening	Odds ratio (95% CI)	*P*
Initial serum amylase				0.007^a^
<100	53 (53.0)	47 (47.0)	1 (reference)	0.007
≥100	23 (31.9)	49 (68.1)	2.40 (1.28–4.52)	
Serum amylase level after 12 h of exposure				<0.001^a^
<100	112 (87.5)	16 (12.5)	1 (reference)	<0.001
≥100	12 (27.3)	32 (72.7)	18.67 (8.02–43.47)	

^a^Chi-square test

Of 172 participants, about 9.3% became dead in the course of treatment. The dead participants had significantly higher mean values of serum amylase levels either within 12 h of exposure (468.60 vs. 135.4 IU/ml, *P*<0.001) or after 12 h of exposure (152.0 vs. 58.9 IU/ml, *P*<0.001) than alive participants (Table 4).

**Table 4:** Relationship of serum amylase levels with outcome of participants with organophosphate poisoning (*N*=172)

	Serum amylase level (mean±SD) (IU/ml)	
Outcome	Within ≤12 h exposure	After 12 h of exposure
Alive (*n*=156)	135.35±78.04	58.89±32.46
Dead (*n*=16)	468.60±53.59	152.00±16.62
*P*	<0.001	<0.001

## Discussions

In our study, patients with initial (within 12 h) and after 12 h of OP poison exposure had more than two-fold and 18-fold higher odds of severe/life-threatening presentations, respectively. In a study from Iran by Zobeiri^11^, the rise in plasma amylase, more than 60% above the normal upper limit on the admission time was associated with clinical severity and mortality following OP poisoning. In a study by Patil *et al*.^7^, from India, amylase levels was raised in all the OP poisoning cases, and the significantly higher mean value of serum amylase was noted in those who became dead than being discharged home. Our study is also consistent with other studies demonstrating that the serum amylase levels rises in OP poisoning and predicts the severity^12^–^14^. Therefore, the serum amylase level in OP poisoning is an easy measurable prognostic marker of OP poisoning^11^,^15^. The possible explanation for the rise in serum amylase in OP poisoning is a pancreatic injury on account of parasympathetic overstimulation and hypersecretion^14^,^16^. In human body, two major organs like pancreas and salivary glands account for the majority of the serum amylase level activity. So, hyperamylasemia is seen in states involving the pancreas and salivary glands.

In OP poisoning, besides hyperamylasemia, there could be many biochemical abnormalities like leukocytosis which could be used for diagnosing and managing the cases^17^,^18^. The OP poisons work by inhibition of cholinesterase enzyme. Henceforth, the estimation of acetylcholinesterase and butyrylcholinesterase are validated screening tools for the diagnosis and prognosis of OP poisoning^19^. Similarly, another potential prognostic indicator in OP poisoning is creatine phosphokinase levels^20^,^21^. The serum creatine phosphokinase levels could rise due to rhabdomyolysis or intermediate syndrome of OP poisoning^22^.

Our study demonstrated higher mean values of serum amylase level either within 12 h of exposure or 12 h postexposure in participants who became dead during the course of treatment. This shows the serum amylase levels dramatically rises in severe/life-threatening presentations with OP poisoning which is evident from multiple other studies^11^,^15^,^22^,^23^.

In our study, among the organophosphorus poisoning, the majority (74%) of the participants with OP poisoning were female. The study by Dash *et al*.^24^, Hussain *et al*.^25^, and Ahmed *et al*.^26^ demonstrated a higher prevalence of OP poisoning in male than females. However, one study have demonstrated a higher preponderance of OP poisoning in female than male^27^. The sex-based predominance of poisoning could differ from place to place, availability of poisons, and multiple factors surrounding them.

In our study, most of the patients (65%) patient did not call for any help and the majority had taken poison for the suicidal purpose (98%). Studies have depicted that suicidal and occupational poisoning to be more common in agricultural workers of developing countries and accidental OP poisoning to be more common in developed countries^26^,^28^,^29^. As agriculture is the main occupation in developing countries like Nepal, pesticides are easily available in lethal and concentrated forms^30^. In our study, ingestion (97%) was the commonest route of poisoning which is common for OP poisoning and are evident from other study^29^.

Our study has also some limitations owing to its cross-sectional nature. We could not figure out the causes of suicidal OP poisoning. We have not studied about the mechanical ventilation and ICU admission effect on serum amylase levels. Thus, a better study design would be required to assess the rise in serum amylase levels with a time of onset of exposure to poison after minimizing the confounding factors. Similarly, a study involving a serial measure of serum amylase following OP poisoning and subsequent mechanical ventilation and/or ICU admission would be of paramount importance.

## Conclusions

The clinical severity of OP poisoning is directly related to serum amylase levels. Importantly, higher mean values of serum amylase levels were depicted in those participants with OP poisoning culminating to death. Thus, serum amylase level could be one of the easy measurable prognostic marker of OP poisonings. Awareness and regulations must be made for its storage and distribution gave the easy route of exposure and higher predominance of suicidal poisoning, especially in young individuals.

## Ethical approval

The ethical clearance was taken from the institutional review board, Maharajgunj Medical Campus, Tribhuwan University Teaching Hospital, Kathmandu, Nepal [Ref. 308 (6-11-E)]. Written informed consent was taken from each participant and the participation was entirely voluntary.

## Consent

Witten informed consent obtained.

## Sources of funding

None.

## Author contribution

B.S., N.J., R.K.M., and R.P.A. conceived and designed the study, with B.S. acting as the guarantor. B.S., G.K.Y., A.R., N.J., B.K.S., N.J., R.P.N., V.B., R.K.M., and R.P.A. performed the literature review. B.S., N.J., B.K.S., N.J., R.P.N., and V.B. contributed to data collection and acquisition. B.S., G.K.Y., N.J., B.K.S., and N.J. performed the data and statistical analysis. B.S., G.K.Y., A.R., N.J., B.K.S., and N.J. wrote the first draft of this manuscript. B.S., G.K.Y., A.R., N.J., B.K.S., N.J., and R.P.N. contributed to the final reviewing and editing of the manuscript before submission to the journal. B.S., R.K.M., and R.P.A. contributed to the critical revisions before approving the final draft of the manuscript for publication. All authors have critically reviewed and approved the final draft and are responsible for the content and similarity index of the manuscript.

## Conflicts of interest disclosure

The authors declare that they have no financial conflict of interest with regard to the content of this report.

## Research registration unique identifying number (UIN)

1. Name of the registry: Research Registry
2. Unique Identifying number or registration ID: researchregistry8675
3. Hyperlink to your specific registration (must be publicly accessible and will be checked): researchregistry8675

## Guarantor

Basudev Subedi.

## Provenance and peer review

Not commissioned, externally peer reviewed.

## Acknowledgements

None.

## References

[1] Sahin I Onbasi K Sahin H . The prevalence of pancreatitis in organophosphate poisonings. Hum Exp Toxicol 2002;21:175–177. 12099618 10.1191/0960327102ht234cr

[2] Singh G Khurana D . Neurology of acute organophosphate poisoning. Neurol India 2009;57:119–125. 19439839 10.4103/0028-3886.51277

[3] Sitaram D . Clinical significance of serum amylase in acute organo phosphorus poisoning; 2016.

[4] Goel A Aggarwal P . Pesticide poisoning. Natl Med J India 2007;20:182–191. 18085124

[5] Chakrabarti K Devkota KC . Retrospective study of suicide cases admitted in Nepal Medical College Teaching Hospital. Nepal Med Coll J 2004;6:116–118. 16295741

[6] Bajracharya SR Prasad PN Ghimire R . Management of organophosphorous poisoning. J Nepal Health Res Counc 2016;14:131–138. 28327676

[7] Patil A Kumar S Inamdar A . Impact of serum amylase level in the outcome of acute organophosphorus poisoning: 2-year cross-sectional study at rural teaching hospital. J Lab Physicians 2022;14:001–005. 10.1055/s-0041-1734015 PMC9519261 36186263

[8] Bardin PG Van Eeden SF . Organophosphate poisoning: grading the severity and comparing treatment between atropine and glycopyrrolate. Crit Care Med 1990;18:956–960. 2394119

[9] Tsai JR Sheu CC Cheng MH . Organophosphate poisoning: 10 years of experience in Southern Taiwan. Kaohsiung J Med Sci 2007;23:112–119. 17389175 10.1016/S1607-551X(09)70385-7 PMC11918116

[10] Mathew G Agha R . STROCSS Group. STROCSS 2021: Strengthening the reporting of cohort, cross-sectional and case-control studies in surgery. Int J Surg 2021;96:106165. 34774726 10.1016/j.ijsu.2021.106165

[11] Zobeiri M . Serum amylase as a prognostic marker of organophosphate poisoning. J Inj Violence Res 2021;13:117–120. 34099593 10.5249/jivr.vo113i2.1632 PMC8435076

[12] Lin CL Yang CT Pan KY . Most common intoxication in nephrology ward organophosphate poisoning. Ren Fail 2004;26:349–354. 15462100 10.1081/jdi-120039816

[13] Aslan S Cakir Z Emet M . Acute abdomen associated with organophosphate poisoning. J Emerg Med 2011;41:507–512. 20850255 10.1016/j.jemermed.2010.05.072

[14] Singh S Bhardwaj U Verma SK . Hyperamylasemia and acute pancreatitis following anticholinesterase poisoning. Hum Exp Toxicol 2007;26:467–471. 17698941 10.1177/0960327107076814

[15] Paul G Kabir MR Kamrul-Hasan ABM . Correlation of serum amylase level with the severity of acute organophosphorus compounds poisoning. Int J Adv Med 2021;8:352–356.

[16] Ahmed A Begum I Aquil N . Hyperamylasemia and acute pancreatitis following organophosphate poisoning. Pak J Med Sci 2009;25:957–961.

[17] Robb EL Baker Erika L. Robb Mari B. Baker . Organophosphate toxicity. StatPearls. StatPearls Publishing; 2022. https://www.ncbi.nlm.nih.gov/books/NBK470430/ 29261901

[18] Kumar S Raisinghani N . Leukocyte count: a reliable marker for the severity of organophosphate intoxication? J Lab Physicians 2018;10:185–188. 29692585 10.4103/JLP.JLP_100_17 PMC5896186

[19] Balali-Mood M Balali-Mood K Moodi M . Health aspects of organophosphorous pesticides in Asian countries. Iran J Public Health 2012;41:1–14. PMC3494223 23304659

[20] Kumar S . Correlation of serum cholinesterase and serum creatine phosphokinase enzymes with the severity and outcome of acute organophosphorus poisoning: study in rural central india; 2016.

[21] Kumar GC Bhuvana K Venkatarathnamma PN . Serum creatine phosphokinase as predictor of intermediate syndrome in organophosphorus poisoning. Indian J Crit Care Med 2015;19:384–387. 26180430 10.4103/0972-5229.160274 PMC4502490

[22] Sumathi ME Kumar SH Shashidhar KN . Prognostic significance of various biochemical parameters in acute organophosphorus poisoning. Toxicol Int 2014;21:167–171. 25253926 10.4103/0971-6580.139800 PMC4170558

[23] Dungdung A Kumar A Kumar B . Correlation and prognostic significance of serum amylase, serum lipase, and plasma cholinesterase in acute organophosphorus poisoning. J Family Med Prim Care 2020;9:1873–1877. 32670933 10.4103/jfmpc.jfmpc_205_20 PMC7346941

[24] Dash SK Mohanty MK Mohanty S . Organophosphorus poisoning: victim specific analysis of mortality and morbidity. Med Sci Law 2008;48:241–245. 18754212 10.1258/rsmmsl.48.3.241

[25] Hussain AM Sultan ST . Organophosphorus insecticide poisoning: management in surgical intensive care unit. J Coll Physicians Surg Pak 2005;15:100–102. 15730836

[26] Ahmed SM Das B Nadeem A . Survival pattern in patients with acute organophosphate poisoning on mechanical ventilation: a retrospective intensive care unit-based study in a tertiary care teaching hospital. Indian J Anaesth 2014;58:11–17. 24700893 10.4103/0019-5049.126780 PMC3968644

[27] Sungur M Güven M . Intensive care management of organophosphate insecticide poisoning. Crit Care 2001;5:211–215. 11511334 10.1186/cc1025 PMC37406

[28] Dharmani C Jaga K . Epidemiology of acute organophosphate poisoning in hospital emergency room patients. Rev Environ Health 2005;20:215–232. 16335577 10.1515/reveh.2005.20.3.215

[29] Adinew GM Asrie AB Birru EM . Pattern of acute organophosphorus poisoning at University of Gondar Teaching Hospital, Northwest Ethiopia. BMC Res Notes 2017;10:149. 28376842 10.1186/s13104-017-2464-5 PMC5381028

[30] Gupta SK Joshi MP . Pesticide poisoning cases attending five major hospitals of nepal. J Nepal Med Assoc 2002;41:447–456.

